# Pedestrian Collision Detection and Avoidance in Cerebral Visual Impairment During Unrestricted Walking in an Immersive Virtual Reality Environment

**DOI:** 10.21203/rs.3.rs-9773075/v1

**Published:** 2026-06-22

**Authors:** Jonathan K. Doyon, Madeleine Heynen, Wei Hau Lew, Alex D. Hwang, Jae-Hyun Jung, Lotfi B. Merabet

**Affiliations:** 1. The Laboratory for Visual Neuroplasticity, Department of Ophthalmology, Massachusetts Eye and Ear, Harvard Medical School, Boston, USA.; 2. Schepens Eye Research Institute of Massachusetts Eye and Ear, Department of Ophthalmology, Harvard Medical School, Boston, MA; 3. NVIDIA Corporation, Westford, MA

**Keywords:** mobility, navigation, cerebral visual impairment, pedestrian collision avoidance, virtual reality, eye tracking

## Abstract

Walking safely through highly crowded environments is a significant challenge for individuals with cerebral visual impairment (CVI). Yet current ophthalmic examinations do not capture functional visual difficulties related to safe mobility. We developed an immersive virtual reality (VR)-based task that tracked eye gaze behaviors within dynamic areas of interest to assess pedestrian collision detection, avoidance, and associated visual scanning in CVI (n=12) compared to control (n=14) participants. Subjects walked through a simulated shopping mall populated with crowds of varying densities. The testing scenario was presented using a head-mounted display with integrated eye tracking, and locomotor, behavioral, and visual scanning responses were recorded. Compared to controls, CVI participants exhibited a slower mean preferred walking speed. They were also less likely and slower to detect target (colliding) pedestrians and were more likely to make a collision. CVI participants were also slower in making their first fixation and followed a larger visual scan path to find the target pedestrian. They also spent more time fixating on non-target compared to target pedestrians. Finally, CVI participants showed greater variability in their performance (including pathing deviations), reflecting a range of individual strategies, and maintained a larger walking safety margin (spatio-temporal envelope). These results provide objective evidence of mobility and associated gaze behaviors in CVI during navigation through highly crowded environments.

## 1. Introduction

Safe mobility in crowded environments relies heavily on processing complex, dynamic visual information. For example, to avoid potential collisions, one must detect and track the trajectories and orientations of surrounding pedestrians, maintain sufficient interpersonal distance and timing, and adjust locomotor behavior in an ongoing perception-action loop (Parisi et al., 2016; Rogge et al., 2021). For individuals living with cerebral visual impairment (CVI), walking and navigating safely through highly crowded environments represents a significant challenge, characterized by difficulties avoiding collisions with other pedestrians and surrounding obstacles (Bennett et al., 2024; Chokron & Dutton, 2023; Dutton et al., 1996; McDowell et al., 2024; St Clair Tracy et al., 2025). Despite the importance of having good mobility skills in crowded environments for personal safety and well-being, developing objective methods to assess pedestrian collision detection and avoidance behaviors in this clinical population has received little attention.

CVI is a brain-based visual disorder caused by an underlying neurological abnormality affecting the development of visual processing pathways and is characterized by a complex spectrum of visual deficits (see (Chang et al., 2024), for a recent definition). Reduced visual acuity and contrast sensitivity, along with visual field defects, are often observed in CVI (Fazzi et al., 2007). In addition, higher-order visual deficits, particularly in relation to visuospatial processing, also represent a core feature of the clinical profile (Boot et al., 2010; Chokron & Dutton, 2023; Dutton et al., 2006; Fazzi et al., 2007; Merabet et al., 2025; Ortibus et al., 2009). Visuospatial processing deficits include difficulties with visual orienting, visual scanning and search, as well as spatial judgment, scene understanding, deploying attention, and tasks requiring visuomotor integration (Ben Itzhak et al., 2023; Lam et al., 2010; Macintyre-Beon, 2010; Zuidhoek et al., 2015). This complex profile of visual challenges can have a profound impact on functional visual abilities and the development of skills related to mobility and independence, including navigating safely in complex, dynamic, and crowded environments (Bennett et al., 2024; Chokron & Dutton, 2023; Dutton et al., 1996; McDowell et al., 2024; St Clair Tracy et al., 2025).

With respect to visuospatial processing abilities, numerous studies have consistently demonstrated evidence of impaired visual search performance in individuals with CVI. Specifically, using a variety of paradigms, CVI participants are less accurate and are slower in finding a target, particularly when searching in visual scenes with high complexity and surrounding clutter (Ben Itzhak et al., 2021; Hokken et al., 2024; Hokken et al., 2025; Kooiker et al., 2015; McDowell & Butler, 2023). In one study carried out by members of our group, visual search performance in CVI was investigated using a desktop virtual reality (VR) environment called the “virtual hallway” (Manley et al., 2022). The task required participants to search and follow the movement of a preselected target (the principal of a fictitious school) walking in a crowd of varying sizes, while visual search performance was monitored using eye tracking. CVI participants were found to be less accurate and were slower in finding the target compared to age-matched controls. Furthermore, search performance in CVI worsened significantly (as indexed by slower response times) with increasing crowd size (Manley et al., 2022). The results of this study provide objective evidence that visual search in CVI related to observing pedestrian movements can be affected by cues and factors related to visual scene complexity and task demands. However, how these visual processing difficulties manifest during active walking in crowded environments, requiring active pedestrian detection and collision avoidance strategies, remains completely unexplored in this population.

In the clinical setting, it is important to highlight that standard ophthalmic examinations typically do not objectively assess or capture the day-to-day functional challenges associated with safe mobility. Furthermore, self-reported difficulties are often overlooked or even dismissed, particularly in individuals with CVI who have visual acuity and visual field measures within the normal or near-normal range (Chandna et al., 2024; van Genderen et al., 2012). Given that safe mobility remains an important skill for independence, there remains an unmet need to develop assessments that can characterize these functional visual abilities in CVI in the context of navigating highly crowded environments.

In this exploratory study, we sought to assess pedestrian collision detection and avoidance, as well as associated gaze behavior, in individuals with CVI compared to controls with neurotypical development. Study participants walked unrestricted through an immersive and realistic VR environment simulating a busy shopping mall with crowds of varying size (viewed with a head-mounted display, or HMD) and were instructed to detect and avoid potential collisions with other pedestrians. The use of immersive VR and an HMD combined with eye-tracking affords numerous advantages for this purpose. First, the high-resolution HMD allows for the display of highly realistic scenarios (including visual and auditory cues), while maintaining a high level of experimental control (e.g., repeated assessments, randomized presentation, and varying task demands) and ensuring participant safety (i.e., minimizing risks related to physical collisions and falls). Furthermore, current HMD designs allow for the capture of multiple objective mobility-related metrics in real-time, including changes in walking speed, target detection and response times, as well as deviations in walking path. Finally, the integration of eye tracking provides further granular assessment of scanning behaviors and visual processing strategies associated with collision detection and avoidance.

In line with previous clinical accounts describing mobility difficulties in CVI, we hypothesized that CVI participants would show a profile of impaired performance on this immersive VR-based task. Specifically, CVI participants would be less likely to detect, slower to respond to, and more likely to collide with target pedestrians compared to controls. CVI participants would also show differences in visual scanning, including a greater magnitude of head rotations, slower time to first fixation on the target colliding pedestrian, longer fixation durations, a higher number of fixations (i.e., counts), and a larger visual scan path while searching for the target. Furthermore, these observed differences in CVI with respect to behavioral and gaze performance would be associated with altered profiles in collision avoidance behavior, including greater pathing deviations and maintaining a larger walking safety margin (as indexed by the spatio-temporal collision envelope; (Hwang et al., 2024; Jung, Hwang, et al., 2023). Finally, consistent with previous observations of visual search behaviors in this population, CVI participants would also show worsening performance with increasing levels of crowd density on this task.

## 2. Methods

### 2.1. Participants

Twelve individuals with CVI (5 females; mean age = 20.75 years ± 4.63 SD; range = 14 to 30 years) were recruited for this study. Another fourteen individuals (9 females; mean age = 23.79 years ± 3.07 SD, range = 16 to 29 years) with normal, or corrected-to-normal, vision and neurotypical development served as comparative controls. The difference in mean age between the two groups was not statistically significant (std. β = −0.74, 95% CI = [−1.51, 0.03], p = 0.574).

CVI participants were all previously diagnosed by an eye care professional with extensive clinical experience in working with this clinical population (see supplementary materials and (Merabet et al., 2023) for further details regarding diagnostic assessment procedures).

Visual-based exclusion criteria included reduced binocular visual acuity precluding the identification of a pedestrian target in isolation, any evidence of oculomotor apraxia (i.e., failure of saccadic initiation), intraocular pathology (other than mild optic atrophy), uncorrected strabismus, and a history of photosensitive epilepsy. Further functional exclusion criteria included poor manual dexterity, severe dyspraxia, or significant impairment with independent ambulation that would preclude participation with the walking demands of the task. Motoric function was categorized using the Manual Ability Classification System (MACS) questionnaire (Eliasson et al., 2006). In this study sample, all the controls and all but two CVI participants were rated as level 1, defined by “handles objects easily and successfully”. Two CVI subjects were rated as level 2, defined by “handles most objects but with some reduced quality and/or speed of achievement” (Eliasson et al., 2006). Based on previously described functional criteria (Dutton & Lueck, 2015), all 12 CVI participants were classified as category 3, defined as having “functionally useful vision and who can work at or near the expected academic level for their age group”.

Underlying causes of CVI included hypoxic ischemia, anoxia, periventricular leukomalacia, structural malformations, as well as genetic and neurometabolic disorders (see Table 1 for complete CVI participant demographics). Neurodevelopmental comorbidities included autism spectrum disorder (ASD), auditory processing disorder (APD), and attention deficit hyperactivity disorder (ADHD). Binocular best-corrected visual acuities (BCVA) ranged from 20/15 to 20/40 Snellen (−0.1 to 0.3 logMAR equivalent). The majority (9 out of 12; 75%) of the CVI participants had BCVAs with the normal to near normal range (i.e., 20/15 to 20/25 Snellen) and all had visual acuity and fixation abilities sufficient for eye tracking calibration and gaze recording (see details regarding visual scanning and gaze metrics below). Half (n=6) of the CVI participants had a documented visual field restriction (3 within the lower visual field, 1 with a left peripheral field defect, 1 with a right homonymous hemianopia, and 1 with a generalized peripheral constriction). All participants walked independently as part of their daily activities. Of the 12 CVI participants, four were regular white cane users, and one used a guide dog to assist with orientation and mobility demands.

Written informed consent was obtained from all subjects and a parent or legal guardian when applicable (i.e., in the case of a minor). The study was approved by the Investigative Review Board at Massachusetts Eye and Ear (Boston, MA, USA) and was conducted according to the tenets of the Declaration of Helsinki.

### 2.2. Apparatus and scenarios

**Figure 1.**
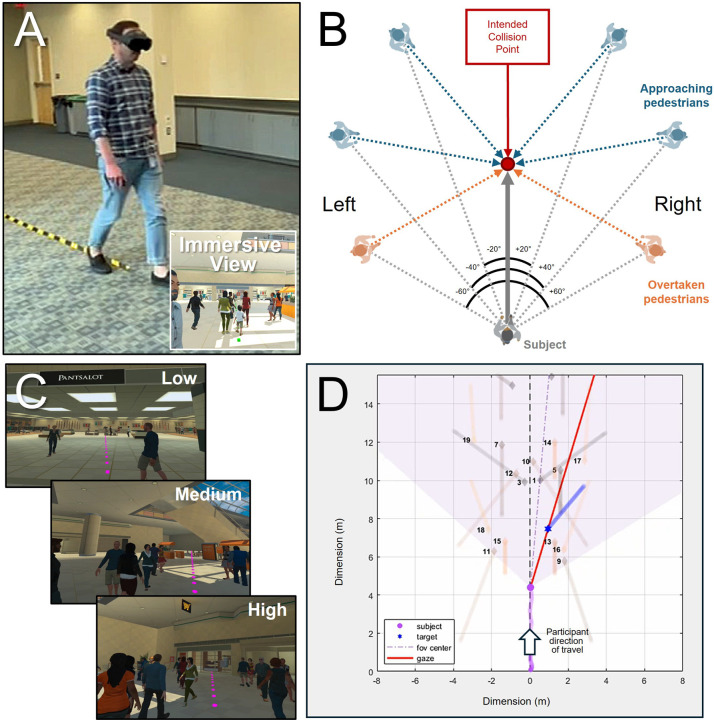
Schematic Depictions of the Experimental Task. (A) The participant walks unrestricted while wearing an HMD that displays an immersive view of the environment (inset figure). (B) Six types of possible colliding targets (only one of which is present in each trial), including approaching and overtaken pedestrians from the left (negative angles) or right (positive angles) side. The colliding pedestrian target was initialized at various locations and walking speeds that maintained a constant time-to-collision (TTC) of 4 seconds and bearing angle (θ) ranging from −60° to +60° in 20° steps. (C) Three crowd density levels were tested: low, medium, and high, corresponding to 1, 10, and 20 noncolliding pedestrians, respectively. (D) Overhead view of logged data (field of view, center of the field of view and head position, and the subject’s gaze on the target are indicated by the light purple cone, dot-dashed line, and solid red line, respectively). The position and walking paths of the target colliding pedestrian (blue star, approaching from the right, +20° direction) and non-colliding pedestrians (other colored symbols, corresponding to a high-density condition) are shown relative to the subject (pink circle).

The VR testing environment simulated a busy shopping mall (including ambient sounds) and was displayed using a Meta Quest Pro HMD with integrated eye tracking (Meta Platforms Inc., Menlo Park, California) with a maximum sampling rate of 90 Hz (Wei et al., 2023) (Figure 1A). The HMD device has an 1800 × 1920-pixel resolution per eye at a 90 Hz refresh rate and approximately 110° binocular field of view (~80° overlap). Based on previous work, the device has a measured average spatial accuracy of 1.65° ± 0.49 SD, spatial precision of 0.69° ± 0.19 SD, and a root-mean-square of 0.84° ± 0.17 SD when used in the unrestrained (i.e., head-free) condition (Wei et al., 2023). During testing, subjects responded to experimental tasks using button presses on the two Meta Quest Pro handheld remote controls (one in each hand) paired to the HMD. Both the HMD and handheld controllers registered 6 degrees of freedom position and rotation. Due to naturally occurring fluctuations in system processing resulting from the many detailed and animated assets present on a given trial, the effective sampling rate varied between 11 and 71 Hz. Subject-level mean effective sampling rates did not differ across groups (std. β = −0.01, SE = 0.031, 95% CI = [−0.11, 0.08], p = 0.766).

All scenarios and assets were developed using the Unity game engine (Unity Technologies, San Francisco, California). A complete description of the VR-simulated environment has been provided previously (see (Jung, Kim, et al., 2023)). Thus, for brevity, details related to the current task design are summarized here. The entire experiment was compiled as a standalone application and installed on the HMD. Each test scenario consisted of a single colliding target pedestrian and a predetermined number of noncolliding walking pedestrians (i.e., non-target distractors). The intended collision point was programmed to occur after a fixed interval and along the participant’s path based on their measured preferred walking speed (PWS; see below) and a corresponding time-to-collision (TTC) of 4 sec. The target pedestrian approached the subject either in a head-on configuration from bearing angles β = ±20° or ±40° relative to the subject or was overtaken by the subject in a side-to-side configuration from β = ±60° (by convention, positive angle values are rightward bearing, and negative angles are leftward bearing; see Figure 1B). Due to the design of the HMD, the pedestrians were not all visible within a single frame of the field of view (thus necessitating visual scanning and head rotations), and their appearance was triggered once the participant started walking. Three levels of crowd density were generated (i.e., low, medium, and high, corresponding to 1, 10, or 20 non-colliding pedestrians, respectively; Figure 1C). The trajectories of the noncolliding pedestrians (serving as non-target distractors) could flank or pass around, but not through the assumed collision point. Multimodal feedback (auditory and haptic on the controller) was used to simulate naturalistic collision consequences when the participant collided with a virtual pedestrian.

Apart from the colliding and non-colliding pedestrians, static standing pedestrians were also present, along with other objects such as furniture and retail stands. Background ambient sounds (e.g., crowd conversations, arcade machines, children playing, etc.) were also presented at a standardized volume to increase the immersive experience of the VR simulation.

### 2.3. Testing Procedure

Before testing, the HMD was adjusted to accommodate the study participant’s inter-pupillary distance (range of 55 to 75 mm) and eye height (i.e., measured from the ground) to ensure optimal viewing clarity, viewpoint, and scaling in VR space. Eye tracking calibration and verification were based on a built-in 9-point program included with the HMD. Subjects briefly fixated a shrinking spherical target (while maintaining their head still) that appeared at 9 locations spatially arranged in a grid and within a visual field spanning approximately 90° (Aziz et al., 2024; Rocchi et al., 2025; Wei et al., 2023).

Testing was carried out along a predefined linear 8 m walking path that was centered within an empty room approximately 13 × 10 m in size. A trial commenced at one end of the walking path and finished at the other end (signaled by a tone). Once the trial was complete, the participant was re-oriented 180° and returned along the same path for the next trial (this cycle was repeated until the end of the testing session). To ensure physical safety, two experimenters were present in the room and monitored the participant during all the walking phases of the task.

To help acclimate the participant to the VR environment and walking scenario, an initial practice session of 12 trials along the same path was completed (empty shopping mall, with no pedestrians present). This was followed by another set of 12 trials to determine the individual’s PWS. For this purpose, participants walked along the same path while viewing the mall surroundings at the medium crowd density level (corresponding to 10 noncolliding pedestrians). Subjects were instructed to walk as they normally would, and their PWS was computed based on their average walking speed across the 12 trials. This measurement of PWS was then used to determine the location of the assumed collision points (based on a TTC of 4 sec) in the main testing scenario.

The main experimental scenario consisted of 21 total trials, 18 of which were collision trials with a target pedestrian, and the remaining 3 trials served as catch trials (i.e., no intended target collision occurred). The 18 collision trials were randomized across the 3 crowd density levels and 6 possible initial bearing angles (3 from each side). The main scenario was then repeated 4 times, for a total of 72 trials per subject. During experimental trials, participants were instructed to walk and visually scan their surroundings naturally and respond (using a button press on the hand-held remote; one in each hand) as soon as they detected a potential collision and indicated from which corresponding side (i.e., left or right). Subjects were instructed to then avoid the potential collision in a natural manner (e.g., slowing down, changing their path heading, sidestepping, etc.).

### 2.4. Outcome Measurements

The Inertia Motion Unit (IMU) incorporated in the HMD recorded the motion and position of the head during each trial, and eye gaze positions were captured through the incorporated eye-tracking functionality (see below). All measurements were logged in real time by the HMD and hand-held remote controls. Head and eye gaze positions (x, y, z; in m) and rotations (φ, θ, ψ; in deg), along with walking speeds (m/s), were logged at each time stamp. Path alterations related to speed, heading, and direction were tracked with the six degrees of freedom data stream generated by translations (x, y, z coordinates) and rotations (φ, θ, ψ Euler angles) of the HMD on each trial (see Figure 1D for an overhead view of an example trial and corresponding logged data).

#### 2.4.1 Preferred Walking Speed

The PWS of each participant was calculated based on the total distance from the beginning to the end of the path (in m) divided by the total time taken (in sec). The final value was computed based on the participant’s average walking speed across 12 trials.

#### 2.4.2 Primary Collision Detection Metrics

Target detection rates and response times were computed as hits/total trials (expressed as a percentage) and the elapsed time between pedestrian onset and the subject’s button response, respectively. Collision events and rates (expressed as a percentage) were determined based on instances of overlapping volumes between the participant’s virtual body and the colliding pedestrian with a 0.25 m radius (based on previous studies, see (Stoudt, 1970)).

#### 2.4.3 Visual Scanning and Gaze Metrics

Gaze metrics were extracted from the three-dimensional rotational time series of the eye (φ, θ, ψ Euler angles) corresponding to the pitch, yaw, and roll, respectively, over the course of each trial. In this testing scenario, a priori-determined areas of interest (AOIs) are ill-suited, given that participants and pedestrians were moving through space. Thus, translations and rotations of the HMD resulted in corresponding transformations of targets of interest within the display. Accordingly, we adopted a dynamic AOI approach in which we tracked the angular size of the pedestrians with respect to the participant and considered the primary AOI the angular span containing the pedestrian at each timestep. To determine whether a fixation occurred, we extracted a series of samples in which the gaze vector formed by the rotational timeseries fell within this AOI. Fixations were defined as instances in which the gaze vector samples remained within the AOI for at least 75% of the total samples recorded within 1 sec of the first sample. From this, we then computed the metrics of time to first fixation, average fixation durations within the AOIs, and average number of fixations. Time to first fixation was measured as the time elapsed from the time of pedestrian onset to the first occurrence of a visual fixation on the target pedestrian. Average fixation duration was measured as the average length of time spent fixating on the target pedestrian within a trial, while average fixation counts were measured based on the sum of the total number of fixations in each trial. Finally, we quantified the extent of the visual scan path by computing the angular displacement of the gaze vector between each time step, based on the equation:

$$\gamma ={\text{cos}}^{-1}\frac{{\vec{V}}_{t-1}\cdot {\vec{V}}_{t}}{\left\|V_{t-1}\right\|\left\|V_{t}\right\|}$$

where ${\vec{V}}_{t-1}$ and ${\vec{V}}_{t}$ are the unit vectors formed by the pitch φ and yaw θ rotations at times t and t-1. We then computed the total angular displacement as the extent of an individual’s scan path trajectory for each trial. All trials were analyzed regardless of whether the colliding pedestrian was successfully detected.

#### 2.4.4 Avoidance Behavior Metrics

Absolute pathing deviation (in m) was computed by the horizontal displacement from the intended path based on the IMU readings obtained from the HMD (Hwang et al., 2024; Jung, Kim, et al., 2023).

As an index of a walking safety margin for collision, we computed the spatio-temporal collision envelope defined as the minimum temporal distance between the participant and the pedestrian (see (Hwang et al., 2024; Hwang et al., 2023; Jung, Hwang, et al., 2023). For this purpose, we traced the minimum instantaneous TTC of the colliding pedestrian projected toward the participant’s location. To determine whether the size of these envelopes differed across groups and density conditions, we then computed the mean area under the curve (auc) value for each crowd density condition.

### 2.5. Analysis

All recorded data on the HMD were then downloaded and processed offline using an in-house Matlab code (version R2021b; Mathworks, Natick, USA) and analyzed using R (ver. 4.4.1; R Foundation for Statistical Computing, Vienna, Austria).

We first examined group differences in PWS, target pedestrian detection rates, response times, and collision rates. Next, we examined visual scanning behaviors extracted from head rotations and gaze metrics corresponding to the target pedestrian. This included time to first fixation, average fixation duration, average fixation counts, and the extent of the visual scan path. Finally, collision avoidance behavior was indexed by group comparisons of pathing deviations and the mean size of the spatio-temporal collision envelopes (based on auc values).

Group comparisons were analyzed using age-adjusted generalized linear mixed effects models as implemented in the {lme4} packages (ver. 1.1–37; (Bates et al., 2015) and {lmerTest} (ver. 3.1–3; (Kuznetsova et al., 2017)) with standardized parameter estimates generated by the {report} package (ver. 0.6.2; (Makowski et al., 2023)). In each model, we selected Gaussian, gamma, binomial, and Poisson error distributions with identity, log, or logit links depending on the nature of the data (e.g., continuous, non-negative, binary, count, etc.). The general specification for each model took the form of:

Outcome~Age+Group×Density+(1∣Subject)+ε

For clarity, we grouped result reporting by the primary factors of interest (i.e., group and density conditions), with group effects reported first, followed by density effects and interactions. Full model results with standardized betas, standard errors, confidence intervals, and p-values are reported in tables S1–13 included with the supplementary materials.

## 3. Results

Overall, participants from both the CVI and control groups were able to perform the task under the immersive VR-simulation and testing conditions. Following test debriefing, all participants reported that they were able to scan, detect, and avoid potential pedestrian collisions with varying levels of difficulty. Furthermore, all participants described the testing scenario as highly realistic and reported that they adopted habitual and naturalistic strategies as they would in the real world. We also noted that performance in the CVI group showed considerable variability across subjects, with some CVI participants performing as well as controls, while others performed considerably worse. Closer examination of the individual CVI participant profiles also revealed a variety of mobility strategies, and selected examples are highlighted as a brief case series comparison (see supplementary materials).

### 3.1 Preferred Walking Speed

**Figure 2.**
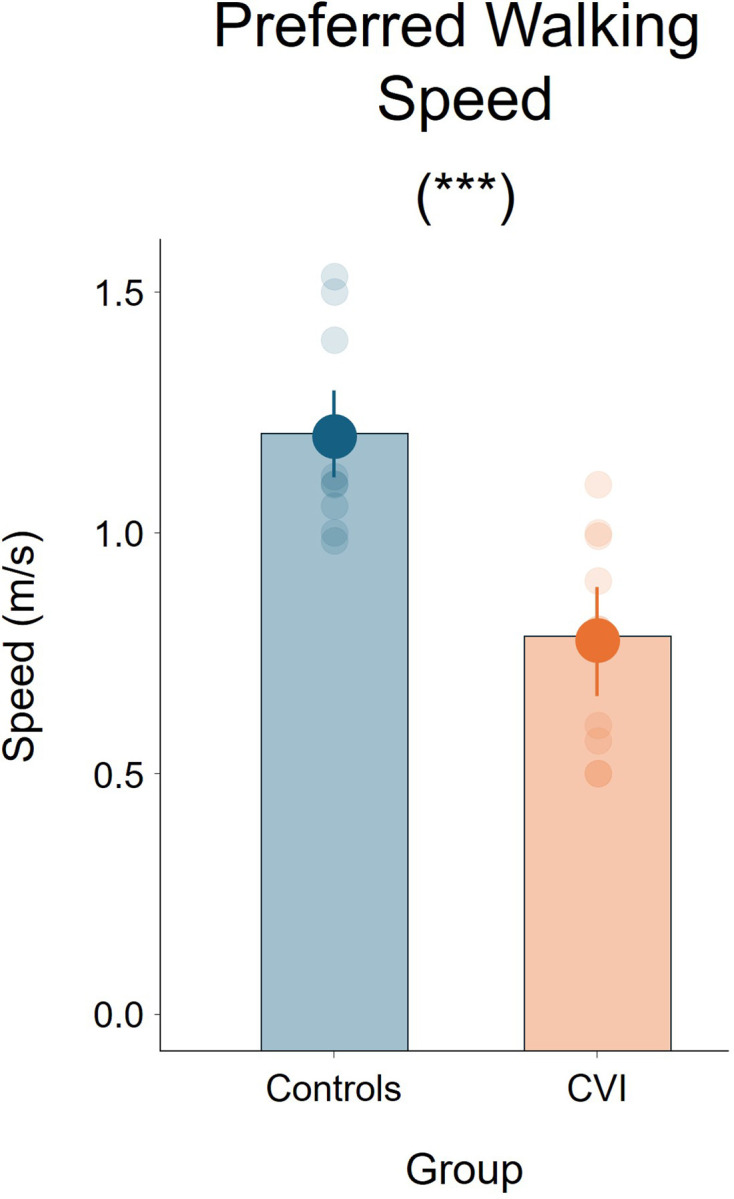
Preferred Walking Speed. Comparing preferred walking speed (PWS) suggests that CVI participants tended to walk more slowly than controls. Bars and large dots correspond to group means, and smaller transparent dots represent individual subject data. Error bars represent 95% confidence intervals. Significant group effects indicated by asterisks enclosed by parentheses, where the significance code corresponds to the following level: *** p < 0.001.

Comparing measured PWS values revealed a significant main effect of group (std. β = −0.43, SE = 0.001, 95% CI = [−0.45, −0.41], p < 0.001; Figure 2), suggesting that overall, the CVI group (mean = 0.80 m/s ± 0.22 SD) adopted a slower baseline walking speed than controls (mean = 1.24 m/s ± 0.17 SD).

### 3.2 Primary Collision Detection Metrics

**Figure 3.**
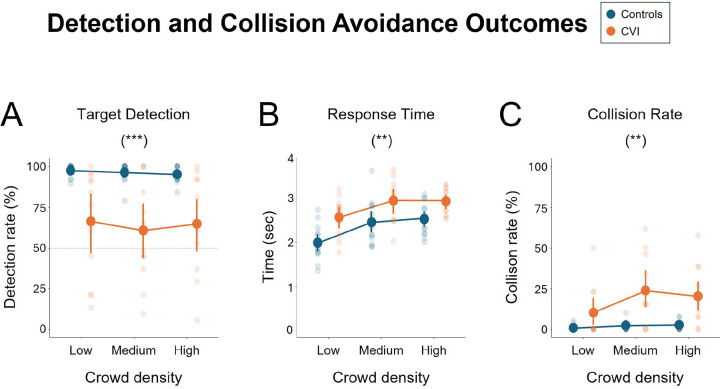
Detection and Collision Avoidance as a Function of Crowd Density. (A) Target detection. CVI participants detected significantly fewer target pedestrians than did controls (B) Response time. CVI participants responded significantly slower to target pedestrians than did controls, and response times were also significantly slower in the medium and high-density conditions compared to the low-density conditions. (C) Collision rate. CVI participants made more collisions with target pedestrians than did controls. Large dots correspond to group means for a given crowd density level, and smaller transparent dots represent individual subject data. Error bars represent 95% confidence intervals. Significant group effects indicated by asterisks enclosed by parentheses, where the significance codes correspond to the following levels: ** p < 0.01; *** p < 0.001.

We found a significant main effect of group with respect to target pedestrian detection (std. β = −2.92, SE = 0.776, 95% CI = [−4.44, −1.40], p = < 0.001; Figure 3A), indicating that CVI participants (mean = 64.80 % ± 47.80 SD) detected fewer colliding pedestrians than controls (mean = 95.60 % ± 20.50 SD). We also found a significant main effect of group regarding response times (std. β = 0.73, SE = 2.66, 95% CI = [0.21, 1.25], p = 0.006; Figure 3B), suggesting that the CVI group (mean = 2.98 sec ± 2.84 SD) responded more slowly than controls (mean = 2.43 sec ± 0.79 SD) when detecting the target pedestrian. Relatedly, we found a significant main effect of group with respect to collision rate (std. β = 2.54, SE = 0.901, 95% CI = [0.77, 4.31], p = 0.005; Figure 3C) suggesting that the CVI group (mean = 18.60 % ± 39.00 SD) was more likely to make a collision with the target pedestrian than controls (mean = 2.30 % ± 15.00 SD).

#### 3.2.1 Effect of Bearing Angle

As part of the initial study design, we manipulated the bearing angle at which the colliding pedestrian was initialized. We then verified whether initial bearing angle had any effect on the three primary collision detection metrics. Statistical analysis revealed no significant effect in terms of collision detection (std. β = −0.19, SE = 0.009, 95% CI = [−0.97, 0.59], p = 0.626) nor with respect to collision rate (std. β = 0.71, SE = 0.837, 95% CI = [−0.93, 2.35], p = 0.396). We did find a small, albeit significant effect of initial bearing angle on response times (std. β = −0.07, SE = 0.016, 95% CI = [−0.10, −0.04], p < 0.001), indicating that responses to targets initialized at larger bearing angles were faster than those at smaller bearing angles (60° mean = 2.38 s ± 3.07 SD; 40° mean = 2.56 s ± 0.93 SD; 20° mean = 2.69 s ± 0.98 SD). Given the size of the observed effect was very small (following Cohen’s recommendations (Cohen, 1988); see discussion for further interpretation), for simplicity, we report all results collapsed across initial bearing angles.

### 3.3 Visual Scanning and Gaze Metrics

**Figure 4.**
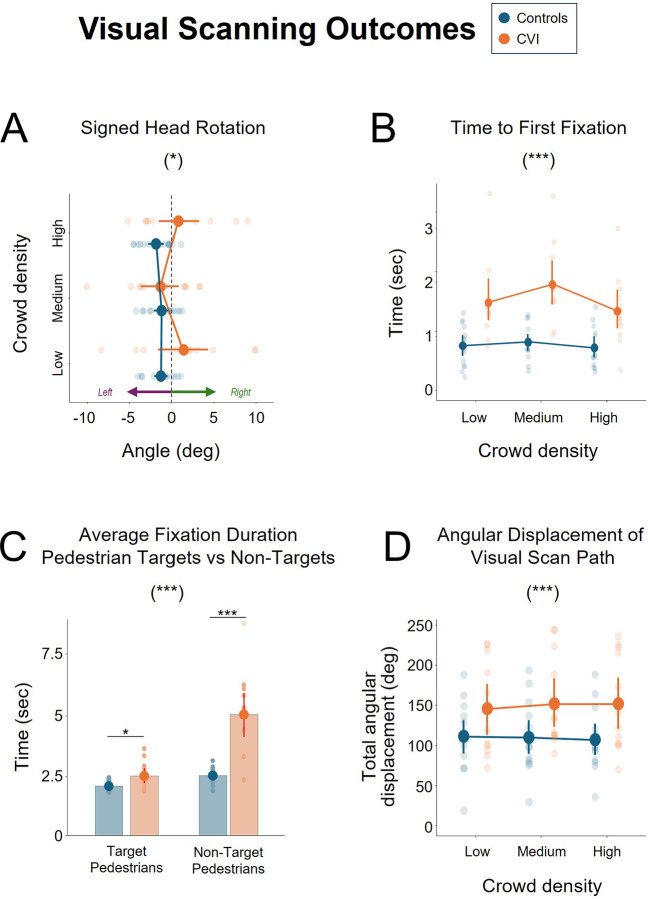
Visual Scanning as a Function of Crowd Density. (A) Signed head rotation. Negative and positive values indicate a leftward and rightward head rotation bias, respectively. Controls showed a slight leftward bias in head rotations, while CVI participants showed greater individual variability. (B) Time to first fixation. CVI participants took significantly longer to fixate the target pedestrian than did controls. (C) Fixation duration as a function of pedestrian type. CVI participants fixated on pedestrians longer than controls, and non-target pedestrians were fixated longer than target pedestrians. CVI participants also fixated on non-target pedestrians longer than target pedestrians. (D) Visual scan path. The extent of the overall visual scan path in CVI participants was larger than in controls. Large dots correspond to group means for a given crowd density, and smaller transparent dots represent individual data. Error bars represent 95% confidence intervals. Significant group effects indicated by asterisks enclosed by parentheses. Significant within-group effects are indicated by asterisk-line pairs. Significance codes correspond to the following levels: * p < 0.05; *** p < 0.001.

Comparing signed head rotations (with rightward and leftward rotations in the horizontal plane indicated by positive and negative values, respectively), we found a significant main effect of group (std. β = 0.26, SE = 1.295, 95% CI = [0.04, 0.49], p = 0.026; Figure 4A) suggesting that controls (mean = −1.79 deg ± 9.09 SD) biased their head slightly leftward compared to the CVI group (mean = 0.065 deg ± 14.04 SD). However, while we observed greater individual variance in the magnitude of signed head rotations in the CVI compared to the control group, comparing the magnitude of absolute head rotations was not statistically significant (CVI mean = 8.24 deg ± 13.82 SD; control mean = 7.57 deg ± 8.65 SD).

Regarding time to first fixation, we observed a significant main effect of group (std. β = 0.20, SE = 0.060, 95% CI = [0.09, 0.32], p = 0.001; Figure 4B) indicating that CVI participants (mean = 1.57 sec ± 1.00 SD) were slower than controls (mean = 0.84 sec ± 0.91 SD) in visually scanning and finding the target colliding pedestrian.

Comparing fixation durations, we found no effect of group when accounting for crowd density (std. β = 0.13, SE = 0.139, 95% CI = −0.20, 0.22], p = 0.932). As a follow-up to this analysis, we also analyzed average fixation duration on non-target (i.e., distractor) pedestrians, where the model took the form of:

FixationDuration~Age+Group×PedestrianType+(1∣Subject)+ε

We found main effects of both group (std. β = 0.17, SE = 0.078, 95% CI = [0.02, 0.32], p = 0.031) and pedestrian type (std. β = 0.18, SE = 0.025, 95% CI = [0.13, 0.22], p < 0.001) indicating that CVI participants (mean = 3.75 sec ± 2.95 SD) fixated on the various pedestrians significantly longer than controls (mean = 2.28 sec ± 1.27 SD) and that non-target pedestrians in general were fixated longer (mean = 3.46 sec ± 2.58 SD) than target pedestrians (mean = 2.16 sec ± 1.45 SD) (Figure 4C). CVI participants also spent significantly more time fixating on non-target pedestrians (mean = 4.88 sec ± 3.25 SD) than on target pedestrians (mean = 2.34 sec ± 1.67 SD; simple contrast: β = 0.689, SE = 0.031, p < 0.001). These effects were subsumed by their interaction (std. β = 0.51, SE = 0.039, 95% CI = [0.44, 0.59], p < 0.001), indicating that while the CVI and control groups fixated target pedestrians for more similar durations (mean difference of 0.30 sec longer for CVI; simple contrast: β = −0.17, SE = 0.078, p = 0.031), the CVI participants fixated the non-target pedestrians for much longer durations than the controls (mean difference = 2.41 sec longer for CVI; simple contrast: β = −0.68, SE = 0.077, p < 0.001).

Regarding the number of fixations, we also failed to find differences across the groups and across the density conditions. We again followed this analysis by including non-target pedestrians, where the model took the form of:

NumberofFixations~Age+Group×PedestrianType+(1∣Subject)+ε)

There was, however, a main effect of pedestrian type (std. β = 0.74, SE = 0.039, 95% CI = [0.66, 0.82], p < 0.001), indicating that non-target pedestrians (mean = 1.98 ± 0.78 SD) were fixated more frequently than target pedestrians (mean = 0.98 ± 0.63 SD).

Finally, comparing the total angular displacement of visual search path, we found a significant main effect of group (std. β = 0.27, SE = 0.059, 95% CI = [0.15, 0.38], p < 0.001; Figure 4D) suggesting that the extent of the overall visual scan path in CVI participants (mean = 143.17 deg ± 96.53 SD) was greater than that of controls (mean = 108.70 deg ± 75.14 SD).

### 3.4 Avoidance Behavior Metrics

**Figure 5.**
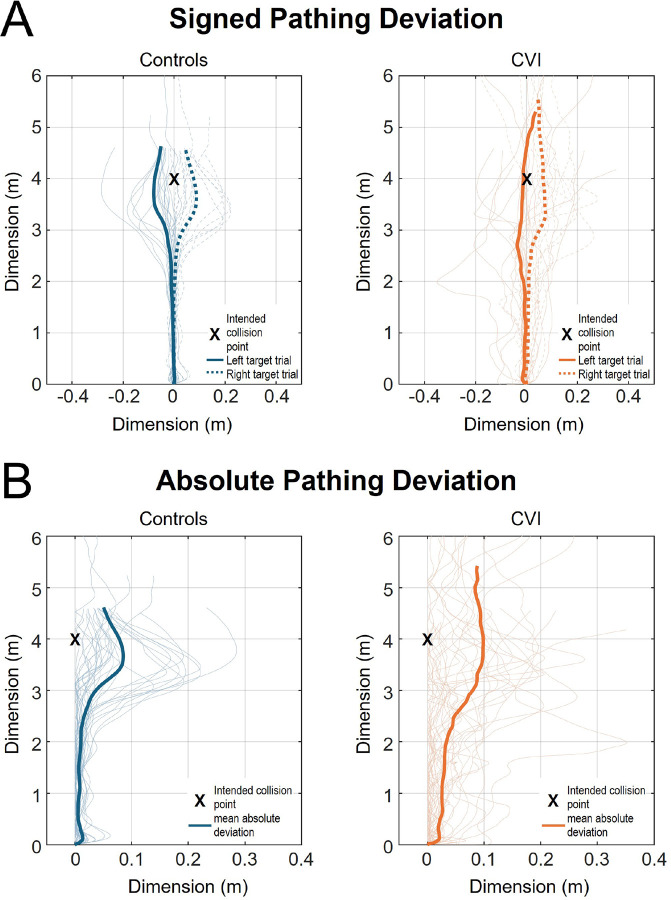
Walking Trajectories and Pathing Deviations. (A) Signed pathing trajectories across all trials and mean pathing deviations in controls (right panel) and CVI (left panel). The solid and dashed lines correspond to trials in which the target was initialized on the left (i.e., θ < 0) and right (i.e., θ > 0) side, respectively. Lighter colored lines represent individual subject data, while darker, thicker lines correspond to group means. Despite CVI participants showing a greater variability in their pattern of signed pathing deviations, these differences did not achieve statistical significance. (B) Absolute pathing deviations indicate the overall magnitude of the lateral deviation collapsed across right and left target initiations. Deviations were significantly smaller in the medium and high-density conditions than in the low-density conditions. Lighter colored lines represent individual subject data, while darker, thicker lines correspond to group means. In all figures, the “x” symbol corresponds to the intended collision point.

Regarding signed and absolute pathing deviations (Figures 5A and 5B), we found no statistically significant effect of group (signed: std. β = 0.001, SE = 0.021, 95% CI = [−0.25, 0.25], p = 0.993; absolute: std. β = 0.29, SE = 0.197, 95% CI = [−0.10, 0.68], p = 0.141) indicating that while the CVI group showed greater variability in pathing. While mean values in pathing deviations tended to be larger in CVI, these differences were not statistically significant between the CVI (signed mean = 0.01 m ± 0.25 SD; absolute mean = 0.13 m ± 0.23 SD) and control (signed mean = −0.002 m ± 0.08 SD; absolute mean = 0.08 m ± 0.05 SD) groups.

**Figure 6.**
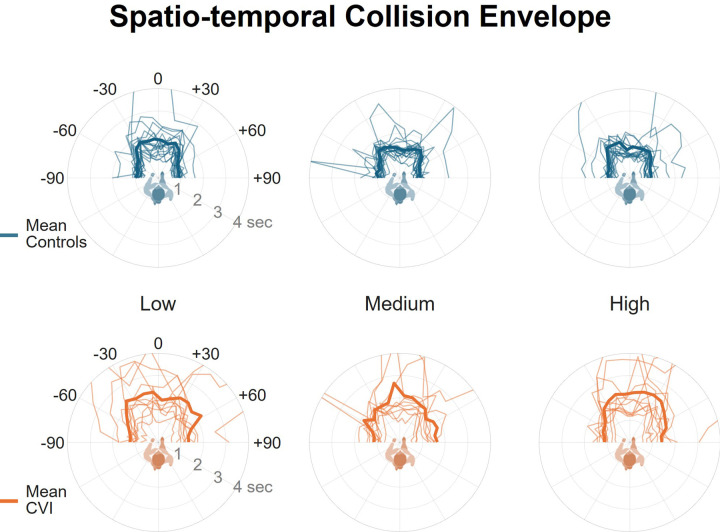
Spatio-temporal Collision Envelope as a Function of Crowd Density. Comparing the spatio-temporal extent of the collision envelope in controls (upper panel) and CVI (lower panel) suggests that overall, CVI participants adopted a larger spatio-temporal collision envelope than did controls. Envelopes in the high-density condition were also significantly smaller than those in the low-density condition. Darker, thicker lines correspond to group means, and light solid lines represent individual subject data. Polar coordinates (r, φ) correspond to the minimum temporal distance (radial coordinate r in seconds) at the bearing angle relative to the participant (angular coordinate φ in degrees) between the participant and the colliding target pedestrian. Note that the r scale is truncated to show variability around the mean, and thus some individual envelopes project beyond the shown r limit.

Comparing spatio-temporal collision envelopes, we found a significant main effect of group (std. β = 0.36, SE = 0.133, 95% CI = [0.10, 0.63], p = 0.006; Figure 6), indicating that CVI participants (mean auc = 5.81 ± 1.92 SD) tended to maintain a larger spatio-temporal area between themselves and the colliding pedestrian (consistent with a larger walking safety margin) than what was observed in the control group (mean auc = 3.66 ± 0.61 SD).

### 3.5 Effect of Manipulating Crowd Size

In general, we found mixed evidence in favor of the hypothesis that varying crowd density would affect overall task performance. In terms of the primary collision detection measures, we did not find a statistically significant effect of crowd density on target detection or collision rates. However, regarding response times, we found main effects for both the medium and high compared to the low crowd density levels (low-medium: std. β = 0.19, SE = 0.022, 95% CI = [0.15, 0.24], p < 0.001; low-high: std. β = 0.25, SE = 0.022, 95% CI = [0.21, 0.29], p < 0.001) indicating that overall, participants responded more slowly at both the medium (mean = 2.73 sec ± 1.08 SD) and high (mean = 2.75 sec ± 1.06 SD) density conditions compared to the low (mean: 2.39 sec ± 2.66 SD) density condition. We also found negative interactions between both medium and high density conditions with group (group × medium: std. β = −0.08, SE = 0.038, 95% CI = [−0.16, −0.003], p = 0.041; group × high: std. β = −0.11, SE = 0.038, 95% CI = [−0.18, −0.03], p = 0.005) suggesting that in both cases, the differences in reaction times between CVI and controls were larger in the low (mean difference = +0.86 sec) density condition compared to both medium (mean difference = +0.37 sec) and high (mean difference = +0.38 sec) density conditions.

In terms of visual scanning and gaze metrics, we found no statistically significant effect of crowd density on the magnitude of head rotations, time to first fixation, visual scan path, or number of fixations. However, regarding fixation duration, we found a significant main effect with the medium compared to the low crowd density condition (std. β = 0.23, SE = 0.064, 95% CI = [0.08, 0.39], p = 0.003) indicating that overall, participants fixated the colliding pedestrian longer in the medium (mean = 2.03 sec ± 0.82 SD) density condition than in the low (mean = 1.79 sec ± 0.74 SD) density condition.

Finally, with respect to avoidance behavior metrics, we found no statistically significant effect of crowd density on signed pathing deviations. However, we found a significant main effect with the high density condition with respect to the spatio-temporal collision envelope (std. β = −0.09, SE = 0.038, 95% CI = [−0.17, −0.01], p = 0.017) suggesting that overall, participants adopted smaller collision envelopes in the high density condition (mean auc = 4.62 ± 1.82 SD) compared to the low density condition (mean auc = 4.75 ± 1.45 SD). We also found significant main effects of both the medium and high density conditions with respect to absolute pathing deviation compared to the low density condition (low-medium: std. β = −0.29, SE = 0.042, 95% CI = [−0.38, −0.21], p < 0.001; low-high: std. β = −0.29, SE = 0.043, 95% CI = [−0.37, −0.20], p < 0.001) indicating that in both cases, participants deviated from the predefined walking path to a lesser extent in the medium (mean = 0.08 m ± 0.07 SD) and high (mean = 0.10 m ± 0.22 SD) than in the low (mean = 0.096 m ± 0.103 SD) density condition. These effects were subsumed by positive interactions of both medium and high density conditions with group (group × medium: std. = 0.22, SE = 0.068, 95% CI = [0.09, 0.36, p = 0.001; group × high: std. β = 0.42, SE = 0.068, 95% CI = [0.29, 0.55], p < 0.001) suggesting that the differences between the CVI and control groups were greater in the medium (mean difference = +0.043 m) and high (mean difference = +0.084 m) density conditions than in the low (mean difference = +0.029 m) density condition.

## 4. Discussion

In this exploratory study, we investigated mobility performance in individuals with CVI by implementing a specially designed immersive VR environment. The use of an HMD enabled the objective assessment of pedestrian detection and collision avoidance behaviors under naturalistic conditions, while captured eye-tracking data provided further insight into visual scanning strategies associated with task performance.

We found experimental support for our overarching hypothesis that, as a group, CVI participants would show a profile consistent with impaired mobility in highly crowded environments compared to controls with neurotypical vision and development. This was supported by 1) a slower preferred walking speed (PWS), 2) fewer correct detections and slower responses to target pedestrians, as well as greater risk of collision, 3) altered visual scanning strategies, including delayed time to first fixation and larger visual scan paths, as well as longer fixation durations on non-target (i.e., distractor) compared to target pedestrians 4) greater variability in pathing deviations and the maintenance of a larger walking safety margin (spatio-temporal collision envelope). CVI participants also showed a wide variety of locomotor and adaptive behaviors to mitigate the risks associated with avoiding pedestrian collisions. Taken together, our observations provide objective evidence of the challenges that individuals with CVI face when interacting and navigating safely in highly crowded environments (Bennett et al., 2024; Chokron & Dutton, 2023; Dutton et al., 1996; McDowell et al., 2024; St Clair Tracy et al., 2025).

Our immersive VR task and experiential approach offered several advantages over traditional methods of assessing mobility performance, such as self-report (Hamed & Masoud, 2023; Tabrett & Latham, 2011), measures of preferred walking speed (PWS; (Chang et al., 2020)), and observations drawn from active ambulation in physical environments (Chang et al., 2020). First, the broad capture of locomotor dynamics and behavioral metrics provided a more detailed analysis of impairment-related differences in mobility performance, under controlled experimental conditions, including the characterization of pedestrian detection and collision avoidance. The recording of head movements and eye gaze patterns also allowed for the analysis of visual scanning behaviors associated with task performance, further supplementing observations drawn from behavioral metrics. Finally, we avoided potential logistical challenges and risks of physical harm (e.g., collisions and falls) that could occur with ambulating in an obstacle course or in real-world settings.

Our work also contributes to a growing body of literature seeking to characterize and objectively assess visual scanning strategies, navigation, and mobility performance in individuals with actual or simulated visual impairments (such as central and peripheral visual field loss; see (David et al., 2020; David et al., 2021; Veerkamp et al., 2025)) and patients with neurological injury (Bowers et al., 2024; de Aquino Costa Sousa et al., 2024; Jung, Kim, et al., 2023; Uimonen et al., 2024). Together, these studies demonstrate the value of developing naturalistic tasks, beyond what is typically assessed during standard ophthalmic examinations, to better understand the impact of visual impairment on real-world activities (Veerkamp et al., 2025).

### 4.1. CVI participants adopted a more cautious walking strategy.

We found that CVI participants adopted a slower mean PWS than controls and tended to show greater variance and modulation in their locomotor dynamics (see also supplementary materials for individual case descriptions). A slower PWS has been observed in individuals with visual impairment (Beggs, 1991; Soong et al., 2004) and is interpreted to signify a more cautious walking behavior and gait pattern, such as reduced speed and step length (Maki, 1997)). Given previous clinical accounts describing difficulties with independent navigation and mobility in CVI, this result is perhaps not surprising. Adopting a slower PWS may prove advantageous for an individual with CVI. For example, walking more slowly is likely to reduce the force of impact from colliding with other pedestrians or obstacles, thereby minimizing the risk of physical harm or injury. Additionally, a slower walking speed (along with the presence of visible mobility aids, such as a white cane) can help signal to others to be more attentive and cautious when approaching an individual with visual impairment.

However, it is noteworthy that a slower PWS was also adopted in CVI participants with visual acuity and visual field function at normal/near normal levels. This suggests that other factors related to the processing of visual information may be contributing to this cautious walking strategy and ultimately, differences in mobility performance in these individuals. Thus, while informative, the measurement of PWS alone does not provide complete insight into why individuals with CVI have challenges with pedestrian detection and collision avoidance. This highlights the value of the multiple behavioral and visual scanning measures captured in this study, which provide further characterization of group-related differences in mobility (see also (Chang et al., 2020) for further discussion on this topic).

### 4.2. CVI participants showed a general profile of impaired pedestrian target detection and collision avoidance.

The CVI group was less likely and slower in detecting target pedestrian collisions compared to controls. This trend appears consistent with the findings of our previous desktop VR study investigating visual search in a scene of walking pedestrians, revealing that CVI participants had significantly lower target detection accuracy and slower response times compared to controls (Manley et al., 2022). Furthermore, as a corollary to the observation of impaired target pedestrian detection, we also observed that CVI participants in this study had a higher rate of collisions, translating (on average) to a 12 to 13 times greater risk. In this context, it is important to consider that this increased likelihood of collision was observed despite the relatively short time scale of the task. Thus, this increased risk could be very disruptive for mobility activities occurring over a much longer period, such as an entire day.

### 4.3. Impaired pedestrian collision detection and avoidance in CVI was associated with differences in visual scanning behavior

Differences in visual scanning behaviors were also observed during this mobility task. While the magnitude of signed head rotations (along the horizontal axis) was not statistically different between groups, CVI participants showed greater variability with respect to varying crowd density. Furthermore, the CVI group was slower in making their first fixation on the target pedestrian. We also observed a greater total angular displacement of visual search paths in CVI, suggesting that these participants searched over a larger spatial extent to find the target pedestrian. This delayed response and larger visual scan path with respect to search behavior are in line with their behavioral profile of slower (manual) response times for target detection.

In contrast, we did not observe statistically significant differences between the CVI and control groups with respect to average fixation durations and number of fixations in response to varying crowd size. However, we did find a significant group difference when considering fixation durations as a function of pedestrian type (i.e., target compared to non-target pedestrians). It is perhaps not surprising that non-target distractor pedestrians would be fixated for longer durations, given that they were more numerous (in the medium and high crowd density levels). However, a significant interaction effect was also observed when further examining pedestrian type. Specifically, on average, the CVI group spent approximately twice as long fixating on non-target pedestrians compared to controls. This differential pattern suggests that CVI participants may have had greater difficulty in selecting unique visual features tied to the target pedestrian and differentiating them from non-target pedestrians and followed a different strategy to make this determination (see below for further discussion).

Another important factor to consider is the potential effect of reduced visual acuity and restrictions in visual field function. Regarding the former, it is possible that increased blur associated with impaired visual acuity in certain CVI participants may have influenced performance. However, the clinical profile of our study participants and results from previous work do not support the view. First, the large majority of the CVI participants (75%) had BCVAs within the normal to near/normal range. Secondly, in our previous desktop VR study, we showed that comparing visual search abilities in CVI participants stratified by normal/near normal and reduced (i.e., worse than 20/30 Snellen) binocular visual acuity showed comparable levels of performance (Manley et al., 2024). Furthermore, work by Chandna and colleagues found that the presence of higher-order visual deficits in CVI was independent of visual acuity levels, as assessed by the Higher Visual Function Questionnaire (HVFQI; (Chandna et al., 2024)) and the ability to integrate complex motion processing signals (Chandna et al., 2021). This suggests that a reduction in visual acuity alone is unlikely to explain the observed difficulties in CVI regarding visual search and overall behavioral performance on this task.

The integrity of visual field function should also be considered, given that a restriction in the extent of the visual scene limits the amount of information that can be processed at a given moment. Indeed, patients with hemianopic visual field loss often report significant challenges with safe mobility and ambulation (de Haan et al., 2015). In a preliminary study, members of our group used a similar immersive VR testing platform to assess pedestrian detection and collision avoidance behaviors in older adults with acquired homonymous hemianopia. Early results suggest that participants with hemifield loss walked more slowly, made fewer detections, were more likely to collide with pedestrians, and biased their head rotations toward the side of the field deficit (Doyon et al., 2025). In contrast to these observations, one of our CVI participants (CVI participant 11; see supplementary materials for further discussion) presented with a dense hemianopia visual field defect and yet was one of our best performers in terms of target pedestrian detection and collision avoidance metrics. This apparent discrepancy may be related to the comparatively younger age of the participant, the early timing of the causative neurological insult, and the presence of highly effective compensatory behaviors based on intensive visual training and therapy. Future studies with larger sample sizes are needed to help disentangle the potential contributory effect of various clinical profile features related to visual functioning, as well as the role of compensatory behaviors and adaptive strategies.

### 4.4. CVI Participants showed differences in collision avoidance behavior related to variations in pathing and maintaining a walking safety margin.

We found that qualitatively, the CVI group showed a high degree of inter-individual variability in terms of the pattern of their pathing deviations. Some participants altered their path early following the onset of the target pedestrian, while others made their pathing deviations much later. At the same time, some made only minimal side-stepping movements while others made wide deviations from the original path. This wide profile also mirrored the greater variance in inter-individual performance observed in CVI with respect to behavioral and visual scanning outcomes. In contrast, control participants typically made only slight lateral pathing deviations combined with a brief slowing and re-establishment of their walking speed to avoid collisions, reflecting a more consistent pattern as a group.

Additionally, CVI participants maintained a larger walking safety margin (as indexed by a greater spatio-temporal envelope) between themselves and the pedestrians. Specifically, the CVI group maintained, on average, a 0.5 to 1 second larger time-to-collision gap with pedestrians compared to controls. This larger safety margin may reflect a more cautious walking strategy and aligns with, but is distinct from, our observation of a slower mean PWS in CVI compared to controls. Given that the computation of the collision envelope incorporates the instantaneous walking speed of the participant, the temporal distance maintained by the participant is not dependent on whether the participant naturally walks faster or slower. Rather, the increased temporal distance likely reflects a more global strategy of cautiousness, which results in CVI participants being more avoidant earlier in time, irrespective of the metric distance that spans the gap between the participant and the pedestrian.

### 4.5. Relation between cautious walking strategy and visual processing deficits in CVI

Taken together, the results from our CVI participants reflect a broad tendency to disengage from other pedestrians when faced with highly crowded environments. Specifically, they tended to decrease their interactions with the surrounding visual elements related to the collision event. The perception and interaction with the complex, unfolding dynamics of the visual scene may be more difficult for individuals with CVI due to underlying deficits related to visuospatial processing, which are commonly present in this population (Chokron & Dutton, 2023; Dutton et al., 2006; Fazzi et al., 2007; Merabet et al., 2025; Ortibus et al., 2009).

Closer examination of our visual stimulus design reveals that the target pedestrian provided unique and strong looming and optical flow signals based on changes in its angular size and differential patterns of global motion relative to the non-target (distractor) pedestrians. Based on the profile of behavioral and visual scanning performance, we surmise that these primary visual signals informing potential collision events were either not identified or not distinguished by our CVI participants. The slower PWS observed in CVI may serve as an adaptive strategy in response to difficulties in extracting these signals. Specifically, a slower PWS reduces the overall speed and magnitude of optic flow patterns generated by self-motion. In turn, patterns of local flow become more salient, potentially making target colliding pedestrians easier to search for and detect. As mentioned earlier, there is evidence that complex motion integration and processing abilities are impaired in CVI (as indexed by higher motion coherence thresholds from viewing psychophysical stimuli such as random dot kinematograms; (Chandna et al., 2021; Merabet et al., 2023; Pamir et al., 2021)). Thus, by slowing down (or even stopping), some CVI participants may be trying to maximize their ability to detect local patterns of optic flow at slower speeds (i.e., the movement of a nearby pedestrian) to recognize a potential nearby collision.

Difficulties in distinguishing the target from surrounding pedestrians may also be explained by impaired higher-order mechanisms related to visual selective attention. Using a variety of visual search paradigms, numerous studies by our group and others have shown that individuals with CVI consistently show decreased accuracy and longer reaction times to find a target. Crucially, performance worsens in the presence of increasing surrounding distractor elements and clutter (Hokken et al., 2024; Hokken et al., 2025; McDowell & Butler, 2023). In one study, Saglam and colleagues carefully analyzed eye-tracking data to characterize gaze pattern sequences in response to two visual search tasks in naturalistic scenes. This analysis revealed that longer response times typically observed in CVI participants signal inefficient search patterns, and are the result of an increased number of fixations, particularly on distractor elements in the display, rather than from slower visual scanning *per se*. Furthermore, recurrent disruptions in maintaining gaze on the target likely reflect difficulties in sustaining attention and the suppression of distractors (Saglam et al., 2026). In the present study, this differential gaze pattern in CVI towards distractors (i.e., non-colliding pedestrians) over the intended target was more closely associated with greater fixation duration than with differences in the number of fixations. Nonetheless, this bias effect toward distractors relative to targets may serve as a hallmark sign explaining impaired visual search abilities in CVI and in relation to commonly observed deficits related to visuospatial processing.

Relatedly, another higher-order mechanism contributing to impaired performance in CVI may be the presence of a narrowed attentional window (Chokron & Dutton, 2023; Das et al., 2007). Using video simulations to explore visual perceptual experiences in CVI, St Clair Tracy and colleagues characterized a pattern of dynamic constriction of their attentional window in response to increased scene complexity. This was particularly evident in the setting of navigating in highly complex and cluttered environments (St Clair Tracy et al., 2025). Thus, a constricted attentional window in the presence of a highly crowded environment may have limited the amount of visual information processed at a given time. Our observation that CVI participants also tended to follow a larger visual scan path to find the target pedestrian also supports this view.

The relationship between the tendency to disengage from other pedestrians and factors associated with underlying visual processing deficits is further illustrated by another of our CVI participants (CVI participant 3; see supplementary materials for further details). This CVI participant dramatically slowed her walking speed at the onset of viewing the surrounding pedestrians, which meant that she did not reach the intended collision point in time, and thus, the collision event never took place. She then attempted to track the path of the pedestrian target, and because of this large pathing deviation, several new unintended collision events resulted as she consequently occupied positions located within the walking paths of the noncolliding pedestrians. This created a “knock-on” effect of cascading collision events that may reflect the real-world risks of hesitating and dramatically slowing walking speed in crowded environments, rendering to an individual with CVI feeling disoriented and overwhelmed.

### 4.6. Changes in Performance in Response to Varying Crowd Size

Based on earlier findings, such as our desktop VR pedestrian visual search study (Manley et al., 2022), we also hypothesized that performance in the CVI group would show a general profile of worsening behavioral and visual scanning performance in response to increasing crowd density. In this study, we found only partial support for this hypothesis. In general, the effects related to increasing crowd density were modest and not statistically robust when comparing the CVI and control groups. For certain outcomes (e.g., target detection and collision rates), differences in performance under the medium and high crowd density conditions were greater than in the low-density conditions, but this modulation effect appeared similar in magnitude for both study groups. This suggests that while increasing task difficulty by manipulating crowd density did lead to worse performance as indexed by certain outcomes, it did not consistently differentiate between the CVI and control groups. The reason for this apparent discrepancy is not clear at this time, but it is likely related to methodological differences such as the visual stimulus display used and overall task design (see below for further discussion).

### 4.7. Study limitations and future directions

This task was primarily focused on mobility; thus, it involved many dimensions related to locomotion, visual processing, as well as sensory-motor integration and coordination abilities. The immersive nature of the task also required visual scanning over a much larger field of view than would be expected from viewing a fixed desktop monitor. Given these design features, we anticipated that CVI participants would find this task more challenging and potentially reveal greater impairment in performance compared to initial observations resulting from our desktop VR pedestrian visual search task (Manley et al., 2022). Indeed, while the study sample was not identical, comparing the primary outcome of target detection (accuracy) in CVI participants from the current and past studies does provide qualitative support for this notion (compare the CVI group mean accuracy score of 64.80% on this study to 88.72% from (Manley et al., 2022)). In contrast, the apparent lack of a robust effect in response to varying crowd density may reflect inherent limitations driven by study design choices and fundamental differences between our immersive walking task and desktop-based viewing.

The most obvious difference between the current and previous studies is that our task required participants to balance both the locomotor demands of walking and collision avoidance, as well as searching and detecting targets in the environment. This increased level of cognitive load may have led participants to prioritize and focus more on certain aspects of the task that otherwise would not be required while viewing a screen-based classical visual search task. Specifically, the sensory-motor coordination demands of our task may have led CVI participants to restrict the degrees of freedom that are typically available during movement and ambulation. While flexible use of these degrees of freedom can allow for adaptive variability and more accurate performance, their constraint can also result in more rigid movements and reduced performance accuracy (Bernshteĭn, 1967; Li, 2006). In this case, CVI participants may have adopted more rigid movement patterns, limiting their ability to adjust their actions and ultimately reducing their success in avoiding collisions.

Technical limitations related to data sampling resolution may have also played a role. The display of fully animated 3D models of pedestrians walking in various configurations placed a computational burden on the HMD capabilities, leading to fluctuations in the actualized sampling rate of captured data. This variability may have reduced our ability to detect finer-grained behaviors that would have otherwise been captured by traditional desktop-based eye trackers capable of higher and more stable sampling rates.

From a study design standpoint, the lack of crowd density-related effects may have been driven by the fact that only a subset of initial bearing angles was chosen. It is possible that the participants were able to implicitly learn where and when to scan to efficiently detect target colliding pedestrians despite the varying numbers of surrounding pedestrians. In addition, all the pedestrians were programmed to walk in a linear path, potentially making it easier to identify and follow their trajectories. Another design factor relates to the relatively narrow temporal window participants had to search, respond, and then avoid the target pedestrian. The intended collision point was calculated and occurred 4 seconds after the target pedestrian appeared, meaning that the participant would have to detect and avoid a potential collision with a narrow temporal window before the trial ended. This may have led to a behavioral strategy that reflected more of a saturation effect, such that the modulation of the participants’ response profile emerged as a dichotomous decision between not crowded (i.e., low density) and very crowded (medium and high density) scenarios. Thus, if we extend the task to play out in a larger physical space, allow for longer walking path durations, incorporate more varying collision events in addition to more granular levels of surrounding pedestrian numbers, we may in turn observe larger group effects with respect to magnitude as well as crowd density.

Despite these limitations, another important observation was the fact that the CVI participants in this study showed greater variability across all outcomes, reflecting a wider distribution of locomotor, pedestrian detection and collision avoidance abilities, as well as visual scanning behaviors. This variability may be related to the underlying heterogeneity of their clinical profile, including the etiology and severity of CVI, the presence of neurodevelopmental comorbidities (e.g., autism spectrum disorder, attention deficit hyperactivity disorder, and cerebral palsy; see Table 1), as well as the degree of confidence with independent mobility. Indeed, some CVI participants performed at levels comparable to those of controls, while others showed clear difficulty by their failure to detect and collisions with nearly all the target pedestrians. This makes drawing generalized conclusions somewhat difficult, and future studies should consider assessing the overall navigation independence and history of orientation and mobility training as part of the clinical profile of the CVI participants. Furthermore, correlational analyses between visual scanning behaviors and overall performance, as well as underlying etiology, should be explored. Should these initial findings be confirmed in a larger study sample, such analyses may help further elucidate associations between the clinical and functional profiles of individuals with CVI in relation to their challenges with mobility.

## 5. Conclusions

The results of the current study suggest that individuals with CVI, while presenting with a wide variance in performance and clinical profile, exhibit significant challenges with pedestrian collision detection and avoidance as assessed with this immersive VR task. Our findings also appear in line with observed higher-order deficits related to visuospatial processing in this clinical population, leading to difficulties with mobility and safe navigation in crowded environments.

## Supplementary Material

Supplementary Files

This is a list of supplementary files associated with this preprint. Click to download.

- Table1CVIParticpantDemographics.docx
- DoyonVRPedestrianSupplementMaterialsFINAL.pdf

Table 1 is available in the Supplementary Files section.

## Data Availability

The data that support the findings of this study are available from the corresponding author upon reasonable request and are subject to IRB approval.
